# Correction: Knowledge, attitudes, and practices associated with vitamin D supplementation: A cross-sectional online community survey of adults in the UK

**DOI:** 10.1371/journal.pone.0314062

**Published:** 2024-11-13

**Authors:** Nuttan Kantilal Tanna, Manisha Karki, Iman Webber, Aos Alaa, Austen El-Osta, Mitch Blair

The fifth author’s name is spelled incorrectly. The correct name is: Austen El-Osta. The correct citation is: Tanna NK, Karki M, Webber I, Alaa A, El-Osta A, Blair M (2023) Knowledge, attitudes, and practices associated with vitamin D supplementation: A cross-sectional online community survey of adults in the UK. PLoS ONE 18(8): e0281172. https://doi.org/10.1371/journal.pone.0281172.
