# Supplementary material for: Knowledge, attitudes, and practices associated with vitamin D supplementation: A cross-sectional online community survey of adults in the UK
Source: PLoS One. 2023 Aug 7;18(8):e0281172. doi: 10.1371/journal.pone.0281172 (PMC10406322; doi:10.1371/journal.pone.0281172)
Supplement: S1 File — (DOCX) [file pone.0281172.s001.docx]

**Supplementary File 1: Online E-Survey Questionnaire**

Facilitators and Barriers in Community supplementation of Vitamin D

**(FABCOM-D)**

Start of Block: QUESTIONS

Imperial College London Department of Primary Care & Public Health is conducting a study to investigate the knowledge and perceptions of the local community regarding vitamin D. This information will hopefully help us make changes to improve your health. The questionnaire should not take you more than **10** minutes. All your responses will be anonymous.

Q1 Are you happy to take part in this study?

- Yes (1)
- No (2)

Q2 Vitamin D (also known as cholecalciferol) is important for health and has been making news headlines recently. Have you heard about vitamin D from any of the following? (Please choose one or more)

- Health professionals (doctor, nurse, dietician, pharmacist) (1)
- Educational institution (school, college, university) (2)
- Media (TV, newspaper, radio, internet, magazine) (3)
- Leaflets/Posters (4)
- Personal research (8)
- General knowledge (9)
- Family/Friends (5)
- I have never heard of vitamin D before! (10)
- Other (please specify) (6) ________________________________________________

Q3 Vitamin D is known to have different health benefits. Which of the following do you think are health benefits of vitamin D? (Please choose one or more)

- Improving intelligence (1)
- Preventing rickets (soft bones in children) (2)
- Improving vision (3)
- Improving hair growth (4)
- Improving skin health (5)
- Preventing osteoporosis (brittle bones) (6)
- Preventing diabetes (high blood sugars) (7)
- Supporting the immune system (10)
- I don't know (8)
- None of the above (9)

Q4 Which of the following do you think can help increase your vitamin D levels in the body? (Please choose one or more)

- Food (1)
- Supplements (2)
- Sunlight (3)
- Exercise (6)
- I don't know (7)

Q5 Which of the following do you think are the top 2 BEST ways to help increase your vitamin D levels?

- Food (1)
- Supplements (2)
- Sunlight (3)
- Exercise (6)
- I don't know (7)

Q6 Which of the following groups of people do you think are more likely to have low levels of vitamin D? (Please choose one or more)

- People who do not spend a lot of time outside during the day (2)
- People who cover up the majority of their skin when they are outside (4)
- People with dark skin (5)
- People who are vegetarian or vegan (6)
- People with some specific medical conditions (for example coeliac disease, liver and kidney problems) (9)
- Children under 4 years (12)
- People over 65 years (13)
- Pregnant women (14)
- None of the above (7)
- I don't know (8)

Q7 Which of the following do you think can affect your vitamin D levels? (Please choose one or more)

- Not spending time outside during the day (4)
- Smoking (7)
- Sunscreen use (8)
- High fat diet (9)
- Not getting enough sunlight (for example because of cloudy weather, air pollution, higher latitudes or winter season) (15)
- Vegetarian or Vegan diet (16)
- None of these (10)
- I don't know (11)

Q8 Current NHS recommendations include taking daily supplements of vitamin D. Do you know what is the recommended daily intake of vitamin D in the UK?

- 10 micrograms (400 IU) (2)
- 25 micrograms (1000 IU) (3)
- 50 micrograms (2000 IU) (4)
- 100 micrograms (4000 IU) (5)
- I don't know (7)
- Other (please state) (6) ________________________________________________

Q9 Do you usually take vitamin D supplements?

- Yes recently (in the last 12 months) (1)
- Yes, for a number of years (4)
- No (2)

Q10 What made you start taking vitamin D supplements? (Please choose one or more)

- A healthcare professional (doctor, nurse, pharmacist) advised me to start taking vitamin D supplements (1)
- Government/ NHS recommendations (9)
- My choice - vitamin D is good for my health (2)
- I don't think I get enough vitamin D from food (3)
- I don't think I get enough sun exposure (4)
- They are part of a multivitamin supplement I take (8)
- A friend or family advised me to take vitamin D supplements (5)
- Other (please state) (6) ________________________________________________

Display This Question:

If Do you usually take vitamin D supplements? = Yes recently (in the last 12 months)

And Do you usually take vitamin D supplements? = Yes, for a number of years

Q11 How do you take your vitamin D supplements?

- I buy them over the counter (1)
- I get them prescribed by my doctor (2)

Display This Question:

If Do you usually take vitamin D supplements? = Yes recently (in the last 12 months)

And Do you usually take vitamin D supplements? = Yes, for a number of years

Q12 What strength of vitamin D supplements do you take?

- 10 micrograms (400 IU) (1)
- 25 micrograms (1000 IU) (2)
- 50 micrograms (2000 IU) (3)
- 100 micrograms (4000 IU) (4)
- Other (please state) (5) ________________________________________________
- I don't know (6)

Display This Question:

If Do you usually take vitamin D supplements? = Yes recently (in the last 12 months)

And Do you usually take vitamin D supplements? = Yes, for a number of years

Q13 On average how often do you take your vitamin D supplement?

- Daily (3)
- Weekly (6)
- Monthly (4)
- I do not take it on a regular basis (please state how often) (5) ___________________________________

Display This Question:

If Do you usually take vitamin D supplements? = Yes recently (in the last 12 months)

And Do you usually take vitamin D supplements? = Yes, for a number of years

Q14 In what form do you take vitamin D? (Please choose one or more)

- Vitamin D capsule (1)
- Vitamin D oil (2)
- Vitamin D drops or spray (3)
- Combined vitamin D & calcium tablet (4)
- Mutlivatamin (5)
- Cod liver oil (6)
- Other (please state) (7) ________________________________________________

Q15 Do you take any other vitamins or supplements?

- Yes (Please state which ones) (1) ________________________________________________
- No (2)

Q16 To your knowledge, have you ever had a blood test to check your vitamin D levels?

- Yes (1)
- No (2)
- I don't know (3)

Q17 In the UK most people are asked to pay for their own vitamin D supplements. To what extent do you agree with the following statements?

|  | Strongly agree (1) | Agree (2) | Neither Agree or Disagree (3) | Disagree (4) | Strongly Disagree (5) |
| --- | --- | --- | --- | --- | --- |
| People at risk of vitamin D deficiency (for example older patients, pregnant women & people with dark skin tones) should have their vitamin D levels checked regularly (7) |  |  |  |  |  |
| People at risk of vitamin D deficiency should get free vitamin D supplements (2) |  |  |  |  |  |
| Doctors should check vitamin D levels before recommending supplements (5) |  |  |  |  |  |
| Testing vitamin D levels should be part of the NHS health check (The NHS health check tests for early signs of heart and brain disease in adults over 40 years) (6) |  |  |  |  |  |
| People should pay for their vitamin D supplements regardless (1) |  |  |  |  |  |

Q18 People take vitamin D either regularly or for a specific time period (for example during the winter). How much are you prepared to pay for a MONTH'S supply of vitamin D supplements?

- Less than £5 (1)
- Between £5-10 (2)
- More than £10 (5)
- I would not be willing to pay to take vitamin D supplements (6)

Q19 If you could obtain them, would you prefer to have foods that are fortified with vitamin D (for example orange juice or milk with added vitamin D) instead of taking separate supplements?

- Yes (1)
- No (2)

Display This Question:

If If you could obtain them, would you prefer to have foods that are fortified with vitamin D (for e... = No

Q20 Why would you not buy foods with added vitamin D? (please choose one or more)

- I don't think they would be safe (1)
- I don't like the idea of eating processed or fortified food (6)
- I don't think I need them (2)
- I don't think I would like their taste (3)
- I think they would be too expensive (4)
- There are not any available in my local supermarket (7)
- Other (please specify) (5) ________________________________________________

Q21 How important are the following factors for you when you decide to take vitamin D supplements?

|  | Very Important (1) | Important (2) | Neutral (3) | Unimportant (4) | Very unimportant (5) |
| --- | --- | --- | --- | --- | --- |
| Knowledge about the health benefits of vitamin D & prevention** (5) |  |  |  |  |  |
| Advice from health professionals (doctors or nurses) & from the NHS (3) |  |  |  |  |  |
| Low vitamin D levels on blood tests (2) |  |  |  |  |  |
| Experiencing symptoms of low vitamin D levels (for example bone pain or muscle weakness) (7) |  |  |  |  |  |
| Reduced exposure to sunlight (15) |  |  |  |  |  |
| Easy access to the supplement (for example at a local supermarket or pharmacy) (12) |  |  |  |  |  |
| Access to the appropriate dosage over the counter (without prescription) (14) |  |  |  |  |  |
| Cost of supplementation (4) |  |  |  |  |  |
| If the supplement contains other vitamins, nutrients & minerals (13) |  |  |  |  |  |
| How often I need to take the supplement (9) |  |  |  |  |  |
| Taste, flavour & smell of supplement (8) |  |  |  |  |  |
| If the supplement is available in the liquid form (11) |  |  |  |  |  |
| How easy it is to chew or swallow the supplement (10) |  |  |  |  |  |

Q22 Which TWO of the following do you find most useful sources for health related information? (Please choose TWO options)

- NHS website (10)
- Health professionals (doctor, nurse, pharmacists) (2)
- Educational institutions (school, college, university) (3)
- Internet/Media (TV, newspaper, radio, internet, magazine) (4)
- Family & friends (8)
- Celebrities & influencers (9)
- Online platforms (for example mums net or Facebook groups) (7)

Q23 10-15 minutes of direct sunlight exposure daily is considered reasonable for promoting healthy levels of vitamin D production.  How many hours a day do you spend on average outside in the sunlight in the spring & summer months?

- Less than 1 hour (6)
- Between 1 - 3 hours (10)
- Between 3 - 5 hours (11)
- More than 5 hours (12)

Q24 On average how much do you cover up during the spring/summer months?

- Minimal coverage (exposure of shoulders and above the knee) (1)
- Moderate coverage (exposure of forearms, below knee and face) (2)
- Maximum coverage (exposure only hands and face) (3)
- Total coverage (no skin exposure) (4)

Q25 Which sex were you assigned at birth?

- Male (1)
- Female (2)
- Other (please specify) (3) ________________________________________________

Display This Question:

If Which sex were you assigned at birth? = Female

Q26 Are you any of the following?

- Menopausal (1)
- Breast-feeding (2)
- Pregnant (3)
- None of the above (4)

Q27 How old are you (years)?

________________________________________________________________

Q28 What is your ethnicity?

- White (1)
- Mixed/Multiple ethnic groups (7)
- Asian/Asian British (2)
- Black/African/Caribbean/Black British (5)
- Other ethnic group (please state) (6) ________________________________________________

Q29 Do you have any dietary restrictions or follow a particular diet?

- Vegetarian (1)
- Vegan (2)
- Lactose intolerant (3)
- Pescatarian (4)
- Kosher (5)
- Halal (6)
- No, I don't follow a particular diet (7)
- Other (please state) (8) ________________________________________________

Q30 Do you have any of the following medical conditions?

- Liver problems (1)
- Kidney problems (2)
- Coeliac disease/Crohn's disease/ulcerative colitis (3)
- No I don't have any of these (4)

Q31 From the skin types (colours) in this picture, which skin type do you think that best describes your skin colour?  

- Type 1 LIGHT, PALE WHITE - Always burns, Never tans (1)
- Type 2 WHITE, FAIR (Usually Burns, Tans with difficulty) (2)
- Type 3 MEDIUM, WHITE TO OLIVE (Sometimes mild burns, gradually tans to Olive (3)
- Type 4 OLIVE, MODERATE BROWN (Rarely burns, Tans with ease to a Moderate Brown) (4)
- Type 5 BROWN, DARK BROWN (Very rarely burns, Tans very easily) (5)
- Type 6 BLACK, VERY DARK BROWN TO BLACK (Never burns, Tans very easily, Deeply pigmented) (6)

Q32 What is your highest level of education?

- Did not finish high school (1)
- High school (2)
- University degree or above (3)
- Other (please specify) (6) ________________________________________________

Q33 Which borough or town in the UK do you live in?

________________________________________________________________

Q34 What is your employment status?

- Employed full time (1)
- Employed part time (2)
- Unemployed (3)
- Unable to work (8)
- Furloughed (4)
- Retired (5)
- Student (6)

Q35 What is your height (in centimetres) and your weight (in kilograms)?

- Height: (4) ________________________________________________
- Weight: (7) ________________________________________________
- Prefer not to say (5)

Thank you for taking the time to answer the questions in this survey.    
Researchers from Imperial College London are looking to interview up to 30 participants (via telephone, Skype, or Microsoft Teams) to learn more about specific themes. Interviews will last 30-45 minutes. Please provide your name & contact details below if this interests you.
We are also happy to answer any questions on the study and can fix a suitable time and date for a chat.

**If you are not able to help at this time, that is also fine. Please click next to register your responses**.

- Name: (1) ________________________________________________
- E-mail: (2) ________________________________________________
- Mobile phone number: (4) ________________________________________________

End of Block: QUESTIONS
