# Supplementary material for: Knowledge, attitudes, and practices associated with vitamin D supplementation: A cross-sectional online community survey of adults in the UK
Source: PLoS One. 2023 Aug 7;18(8):e0281172. doi: 10.1371/journal.pone.0281172 (PMC10406322; doi:10.1371/journal.pone.0281172)
Supplement: S2 File — (DOCX) [file pone.0281172.s002.docx]

**Supplementary File 2 : Summary Document Tables - Facilitators and Barriers with Community supplementation of Vitamin D (FABCOM-D)**

**Objective**

To assess the knowledge, attitude and practices (KAP) of the general public and identify barriers and facilitators that drive routine vitamin D supplementation in the community setting.

**Total Respondents** (557-71) = **486**

**TABLE 1:**

|  | N | (%) |
| --- | --- | --- |
| **PARTICIPANT DEMOGRAPHIC** | | |
|  | | |
| **Gender** | | |
| Female | 130 | (26.7) |
| Male | 356 | (73.3) |
| **(Female)** |  |  |
| Menopausal | 116 | (32.8) |
| Breast-feeding | 1 | (0.3) |
| Pregnant | 4 | (1.1) |
| None of the above | 233 | (65.8) |
| **Age** | | |
| 20-30 | 51 | (10.5) |
| 31-40 | 89 | (18.3) |
| 41-50 | 74 | (15.2) |
| 51-60 | 136 | (28.0) |
| 61-70 | 115 | (23.7) |
| 71 and Over | 21 | (4.3) |
| **Ethnicity** | | |
| White | 239 | (49.2) |
| Mixed/Multiple ethnic groups | 14 | (2.9) |
| Asian/Asian British | 135 | (27.8) |
| Black/African/Caribbean/Black British | 78 | (16.0) |
| Other | 63 | (13.0) |
| **Education** | | |
| Did not finish high school | 8 | (1.6) |
| High school | 66 | (13.6) |
| University degree or above | 372 | (76.5) |
| Other (please specify) | 40 | (8.2) |
| **Employment** | | |
| Employed full time | 257 | (53.0) |
| Employed part time | 101 | (20.8) |
| Unemployed | 11 | (2.3) |
| Furloughed | 4 | (0.8) |
| Retired | 78 | (16.1) |
| Student | 23 | (4.7) |
| Unable to work | 11 | (2.3) |
| **What is your height (in centimetres) and your weight (in kilograms)?** | | |
|  |  |  |
| **Dietary Restrictions** | | |
| Vegetarian | 80 | (16.5) |
| Vegan | 14 | (2.9) |
| Lactose intolerant | 13 | (2.7) |
| Pescatarian | 13 | (2.7) |
| Kosher | 6 | (1.2) |
| Halal | 50 | (10.3) |
| No, I don't follow a particular diet | 275 | (56.6) |
| Other (please state) | 35 | (7.2) |
| **Medical conditions** | | |
| Liver problems | 11 | (2.3) |
| Kidney problems | 43 | (9.0) |
| Coeliac disease/Crohn's disease/ulcerative colitis | 12 | (2.5) |
| No I don't have any of these | 412 | (86.2) |
| **From the skin types (colours) in this picture, which skin type do you think that best describes your skin colour?** | | |
| Type 1 LIGHT, PALE WHITE - Always burns, Never tans | 39 | (8.0) |
| Type 2 WHITE, FAIR (Usually Burns, Tans with difficulty) | 117 | (24.1) |
| Type 3 MEDIUM, WHITE TO OLIVE (Sometimes mild burns, gradually tans to Olive | 141 | (29.0) |
| Type 4 OLIVE, MODERATE BROWN (Rarely burns, Tans with ease to a Moderate Brown) | 96 | (19.8) |
| Type 5 BROWN, DARK BROWN (Very rarely burns, Tans very easily) | 69 | (14.2) |
| Type 6 BLACK, VERY DARK BROWN TO BLACK (Never burns, Tans very easily, Deeply pigmented) | 24 | (4.9) |
| **Which borough or town in the UK do you live in?** | | |
|  | | |
|  | | |
| **Q2:**  **Vitamin D (also known as cholecalciferol) is important for health and has been making news headlines recently. Have you heard about vitamin D from any of the following? (Please choose one or more)** | | |
| Health professionals (doctor, nurse, dietician, pharmacist) | 302 | (62.3) |
| Educational institution (school, college, university) | 106 | (21.9) |
| Media (TV, newspaper, radio, internet, magazine) | 207 | (42.7) |
| Leaflets/Posters | 42 | (8.7) |
| Personal research | 141 | (29.1) |
| General knowledge | 362 | (74.6) |
| Family/Friends | 218 | (44.9) |
| I have never heard of vitamin D before! | 5 | (1.0) |
| Other (please specify) | 14 | (2.9) |
| **Q3: Vitamin D is known to have different health benefits. Which of the following do you think are health benefits of vitamin D? (Please choose one or more)** | | |
| Improving intelligence | 27 | (5.6) |
| Preventing rickets (soft bones in children) | 308 | (63.5) |
| Improving vision | 47 | (9.7) |
| Improving hair growth | 98 | (20.2) |
| Improving skin health | 155 | (32.0) |
| Preventing osteoporosis (brittle bones) | 379 | (78.1) |
| Preventing diabetes (high blood sugars) | 44 | (9.1) |
| Supporting the immune system | 399 | (82.3) |
| I don't know | 6 | (1.2) |
| None of the above | 1 | (0.2) |
| **Q4: Which of the following do you think can help increase your vitamin D levels in the body? (Please choose one or more)** | | |
| Food | 369 | (75.9) |
| Supplements | 430 | (88.5) |
| Sunlight | 480 | (98.8) |
| Exercise | 53 | (10.9) |
| **Q5: Which of the following do you think are the top 2 BEST ways to help increase your vitamin D levels?** | | |
| Food | 187 | (38.5) |
| Supplements | 338 | (69.5) |
| Sunlight | 456 | (93.8) |
| Exercise | 14 | (2.9) |
| I don't know | 1 | (0.2) |
| **Q6: Which of the following groups of people do you think are more likely to have low levels of vitamin D? (Please choose one or more)** | | |
| People who do not spend a lot of time outside during the day | 421 | (86.6) |
| People who cover up the majority of their skin when they are outside | 367 | (75.5) |
| People with dark skin | 307 | (63.2) |
| People who are vegetarian or vegan | 158 | (32.5) |
| People with some specific medical conditions (for example coeliac disease, liver and kidney problems) | 211 | (43.4) |
| Children under 4 years | 94 | (19.3) |
| People over 65 years | 228 | (46.9) |
| Pregnant women | 156 | (32.1) |
| None of the above | 1 | (0.2) |
| I don't know | 8 | (1.6) |
| **Q7: Which of the following do you think can affect your vitamin D levels? (Please choose one or more)** | | |
| Not spending time outside during the day | 398 | (81.9) |
| Smoking | 84 | (17.3) |
| Sunscreen use | 150 | (30.9) |
| High fat diet | 51 | (10.5) |
| Not getting enough sunlight (for example because of cloudy weather, air pollution, higher latitudes or winter season) | 439 | (90.3) |
| Vegetarian or Vegan diet | 151 | (31.1) |
| I don't know | 8 | (1.6) |
| **Q8: Current NHS recommendations include taking daily supplements of vitamin D. Do you know what is the recommended daily intake of vitamin D in the UK?** | | |
| 10 micrograms (400 IU) | 164 | (33.8) |
| 25 micrograms (1000 IU) | 130 | (26.8) |
| 50 micrograms (2000 IU) | 30 | (6.2) |
| 100 micrograms (4000 IU) | 42 | (8.7) |
| Other (please state) | 5 | (1.0) |
| I don't know | 114 | (23.5) |
| **Q9: Do you usually take vitamin D supplements?** | | |
| Yes recently (in the last 12 months) | 205 | (42.2) |
| No | 99 | (20.4) |
| Yes, for a number of years | 182 | (37.4) |
| **Q10: What made you start taking vitamin D supplements? (Please choose one or more)** | | |
|  | | |
| **Q11: How do you take your vitamin D supplements?** | | |
|  | | |
| **Q12: What strength of vitamin D supplements do you take?** | | |
|  | | |
| **Q13: On average how often do you take your vitamin D supplement?** | | |
|  | | |
| **Q14: In what form do you take vitamin D? (Please choose one or more)** | | |
|  | | |
| **Q15: Do you take any other vitamins or supplements?** | | |
| Yes (Please state which ones) | 282 | (58.1) |
| No | 203 | (41.9) |
| **Q16: To your knowledge, have you ever had a blood test to check your vitamin D levels?** | | |
| Yes | 251 | (51.6) |
| No | 193 | (39.7) |
| I don't know | 42 | (8.6) |
| **Q17: In the UK most people are asked to pay for their own vitamin D supplements. To what extent do you agree with the following statements?** | | |
| **People at risk of vitamin D deficiency (for example older patients, pregnant women & people with dark skin tones) should have their vitamin D levels checked regularly** | | |
| Agree | 388 | (80.2) |
| Neither agree nor disagree | 51 | (10.5) |
| Disagree | 45 | (9.3) |
| **People at risk of vitamin D deficiency should get free vitamin D supplements** | | |
| Agree | 361 | (75.7) |
| Neither agree nor disagree | 76 | (15.9) |
| Disagree | 40 | (8.4) |
| **Doctors should check vitamin D levels before recommending supplements** | | |
| Agree | 378 | (80.8) |
| Neither agree nor disagree | 58 | (12.4) |
| Disagree | 32 | (6.8) |
| **Testing vitamin D levels should be part of the NHS health check (The NHS health check tests for early signs of heart and brain disease in adults over 40 years** | | |
| Agree | 425 | (91.2) |
| Neither agree nor disagree | 26 | (5.6) |
| Disagree | 15 | (3.2) |
| **People should pay for their vitamin D supplements regardless** | | |
| Agree | 72 | (15.5) |
| Neither agree nor disagree | 149 | (32.1) |
| Disagree | 243 | (52.4) |
| **Q18: People take vitamin D either regularly or for a specific time period (for example during the winter). How much are you prepared to pay for a MONTH'S supply of vitamin D supplements?** | | |
| Less than £5 | 241 | (51.5) |
| Between £5-10 | 168 | (35.9) |
| More than £10 | 20 | (4.3) |
| I would not be willing to pay to take vitamin D supplements | 39 | (8.3) |
| **Q19: If you could obtain them, would you prefer to have foods that are fortified with vitamin D (for example orange juice or milk with added vitamin D) instead of taking separate supplements?** | | |
| Yes | 298 | (61.6) |
| No | 186 | (38.4) |
| **Q20: Why would you not buy foods with added vitamin D? (please choose one or more)** | | |
| I don't think they would be safe | 6 | (3.3) |
| I don't like the idea of eating processed or fortified food | 104 | (56.5) |
| I don't think I need them | 13 | (7.1) |
| I don't think I would like their taste | 12 | (6.5) |
| I think they would be too expensive | 39 | (21.2) |
| There are not any available in my local supermarket | 16 | (8.7) |
| Other (please specify) | 53 | (28.8) |
| **Q21: How important are the following factors for you when you decide to take vitamin D supplements?** | | |
| **Knowledge about the health benefits of vitamin D & prevention** | | |
| Important | 473 | (97.3) |
| Neutral | 12 | (2.5) |
| Unimportant | 1 | (0.2) |
| **Advice from health professionals (doctors or nurses) &amp; from the NHS** | | |
| Important | 437 | (91.8) |
| Neutral | 32 | (6.7) |
| Unimportant | 7 | (1.5) |
| **Low vitamin D levels on blood tests** | | |
| Important | 434 | (92.5) |
| Neutral | 24 | (5.1) |
| Unimportant | 11 | (2.3) |
| **Experiencing symptoms of low vitamin D levels (for example bone pain or muscle weakness)** | | |
| Important | 432 | (91.9) |
| Neutral | 28 | (6.0) |
| Unimportant | 10 | (2.1) |
| **Reduced exposure to sunlight** | | |
| Important | 389 | (85.3) |
| Neutral | 56 | (12.3) |
| Unimportant | 11 | (2.4) |
| **Easy access to the supplement (for example at a local supermarket or pharmacy** | | |
| Important | 406 | (88.1) |
| Neutral | 41 | (8.9) |
| Unimportant | 14 | (3.0) |
| **Access to the appropriate dosage over the counter (without prescription)** | | |
| Important | 426 | (91.6) |
| Neutral | 34 | (7.3) |
| Unimportant | 5 | (1.1) |
| **Cost of supplementation** | | |
| Important | 384 | (83.3) |
| Neutral | 62 | (13.4) |
| Unimportant | 15 | (3.3) |
| **If the supplement contains other vitamins, nutrients & minerals** | | |
| Important | 247 | (53.6) |
| Neutral | 151 | (32.8) |
| Unimportant | 63 | (13.7) |
| **How often I need to take the supplement** | | |
| Important | 331 | (72.3) |
| Neutral | 84 | (18.3) |
| Unimportant | 43 | (9.4) |
| **Taste, flavour &amp; smell of supplement** | | |
| Important | 207 | (45.2) |
| Neutral | 155 | (33.8) |
| Unimportant | 96 | (21.0) |
| **If the supplement is available in the liquid form** | | |
| Important | 103 | (22.6) |
| Neutral | 166 | (36.4) |
| Unimportant | 187 | (41.0) |
| **How easy it is to chew or swallow the supplement** | | |
| Important | 207 | (45.1) |
| Neutral | 143 | (31.2) |
| Unimportant | 109 | (23.7) |
| **Q22: Which TWO of the following do you find most useful sources for health related information? (Please choose TWO options)** | | |
| NHS website | 343 | (71.2) |
| Health professionals (doctor, nurse, pharmacists) | 371 | (77.0) |
| Educational institutions (school, college, university) | 66 | (13.7) |
| Internet/Media (TV, newspaper, radio, internet, magazine) | 152 | (31.5) |
| Family & friends | 63 | (13.1) |
| Celebrities & influencers | 5 | (1.0) |
| Online platforms (for example mums net or Facebook groups) | 27 | (5.6) |
| **Q23: 10-15 minutes of direct sunlight exposure daily is considered reasonable for promoting healthy levels of vitamin D production. How many hours a day do you spend on average outside in the sunlight in the spring & summer months?** | | |
| Less than 1 hour | 185 | (38.1) |
| Between 1 - 3 hours | 241 | (49.6) |
| Between 3 - 5 hours | 46 | (9.5) |
| More than 5 hours | 14 | (2.9) |
| **Q24: On average how much do you cover up during the spring/summer months?** | | |
| Minimal coverage (exposure of shoulders and above the knee) | 83 | (17.1) |
| Moderate coverage (exposure of forearms, below knee and face) | 335 | (68.9) |
| Maximum coverage (exposure only hands and face) | 66 | (13.6) |
| Total coverage (no skin exposure) | 2 | (0.4) |
