# Supplementary material for: Knowledge, attitudes, and practices associated with vitamin D supplementation: A cross-sectional online community survey of adults in the UK
Source: PLoS One. 2023 Aug 7;18(8):e0281172. doi: 10.1371/journal.pone.0281172 (PMC10406322; doi:10.1371/journal.pone.0281172)
Supplement: S3 File — (DOCX) [file pone.0281172.s003.docx]

**Supplementary File 3:**

**Table 4: Univariable and multivariable association of demographic characteristics with Vit-D intake.**

|  | **Vit-D supplement intake** | | | |
| --- | --- | --- | --- | --- |
|  | **Univariable model** | | **Multivariable model** | |
|  | **OR (95% CI)** | **p-value** | **adj.OR (95% CI)** | **p-value** |
| **Age** | 0.71 (0.61, 0.84) | <0.01 | 0.70 (0.59, 0.83) | <0.01 |
| **Gender** |  |  |  |  |
| Female | Ref. |  | Ref. |  |
| Male | 0.63 (0.39, 1.01) | 0.05 | 0. .65 (0.40, 1.06) | 0.09 |
| **Ethnicity** |  |  |  |  |
| White | Ref. |  | Ref. |  |
| Mixed/Multiple ethnic groups | 0.84 (0.29, 2.47) | 0.76 | 1.30 (0.43, 3.94) | 0.64 |
| Asian/Asian-British | 0.95 (0.30, 2.94) | 0.93 | 1.29 (0.40, 4.14) | 0.65 |
| Black/African/Caribbean | 1.16 (0.41, 3.23) | 0.77 | 1.84 (0.63, 5.34) | 0.26 |

- Logistic regression was carried out to assess the effect of age, gender, and ethnicity on Vit-D intake. After adjusting gender and ethnicity, results suggest that with every one-unit increase in age, there is likely to be a 30% decrease in the Vit-D supplement intake (adj.OR=0.70, 95% CI (0.59 to 0.83).
- The univariable and multivariable models do not indicate any significant associations between different ethnicities (Mixed/Multiple ethnic groups, Asian/Asian-British, Black/African/Caribbean) and vitamin D supplement intake. The odds ratios for these ethnic groups are close to 1, suggesting no substantial differences in the odds of supplement intake compared to the reference group (White). The corresponding p-values are all above 0.05.
