# Supplementary material for: Knowledge, attitudes, and practices associated with vitamin D supplementation: A cross-sectional online community survey of adults in the UK
Source: PLoS One. 2023 Aug 7;18(8):e0281172. doi: 10.1371/journal.pone.0281172 (PMC10406322; doi:10.1371/journal.pone.0281172)
Supplement: Attachment — Submitted filename: KAP Vit D.docx [file pone.0281172.s004.docx]

Knowledge, attitudes, and practices associated with Vitamin D supplementation: A cross-sectional online community survey of adults in the UK

Reviewer’s comments

1. The study objective is about barriers and drivers of vitamin D supplementation in defined population. The result is more focused towards association of risk factor to vitamin D usage (table 2). Either this should be added in the objectives or deleted from the results.
2. It will be more impactful if a table is added showing which barriers and which drivers were identified for vitamin D usage in the study population.
